# Glyphosate induces immune dysregulation in honey bees

**DOI:** 10.1186/s42523-022-00165-0

**Published:** 2022-02-22

**Authors:** Erick V. S. Motta, J. Elijah Powell, Nancy A. Moran

**Affiliations:** Department of Integrative Biology, University of Texas at Austin, 2506 Speedway, Austin, TX 78712 USA

**Keywords:** Honey bees, Glyphosate, Tylosin, Humoral immunity, Cellular immunity, Microbiome

## Abstract

**Background:**

Similar to many other animals, the honey bee *Apis mellifera* relies on a beneficial gut microbiota for regulation of immune homeostasis. Honey bees exposed to agrochemicals, such as the herbicide glyphosate or antibiotics, usually exhibit dysbiosis and increased susceptibility to bacterial infection. Considering the relevance of the microbiota–immunity axis for host health, we hypothesized that glyphosate exposure could potentially affect other components of the honey bee physiology, such as the immune system.

**Results:**

In this study, we investigated whether glyphosate, besides affecting the gut microbiota, could compromise two components of honey bee innate immunity: the expression of genes encoding antimicrobial peptides (humoral immunity) and the melanization pathway (cellular immunity). We also compared the effects of glyphosate on the bee immune system with those of tylosin, an antibiotic commonly used in beekeeping. We found that both glyphosate and tylosin decreased the expression of some antimicrobial peptides, such as apidaecin, defensin and hymenoptaecin, in exposed honey bees, but only glyphosate was able to inhibit melanization in the bee hemolymph.

**Conclusions:**

Exposure of honey bees to glyphosate or tylosin can reduce the abundance of beneficial gut bacteria and lead to immune dysregulation.

**Supplementary Information:**

The online version contains supplementary material available at 10.1186/s42523-022-00165-0.

## Background

A well-balanced gut microbiota is usually associated with positive benefits to host health including digestion of recalcitrant components of the diet, production of nutrients (e.g., short-chain fatty acids), regulation of immune homeostasis and protection against opportunistic pathogens [1]. Therefore, imbalances to the gut microbiota, commonly called dysbiosis, may lead to various negative consequences to hosts, culminating in poor development, immune dysregulation and disease [2]. These effects occur in many animals, including the Western honey bee, *Apis mellifera*, an important agricultural pollinator that relies on a beneficial gut microbiota to maintain homeostasis [3–5]. The honey bee gut microbiota is dominated by 5 to 8 host-restricted bacterial lineages; this community fully colonizes the bee gut within 5 days after emergence and remains compositionally stable until a bee become a forager and is exposed to environmental microbes [3]. At emergence from the pupal stage, the bee digestive tract is devoid of microbes [6]. Experiments using gnotobiotic honey bees have demonstrated the importance of the native gut microbiota, including the contribution of specific bacterial members, such as *Snodgrassella alvi* and *Frischella perrara*, to the stimulation and regulation of the immune system [7–9].

Honey bees have a robust innate immune system that, along with physical barriers (e.g., exoskeleton cuticle, peritrophic membranes lining the midgut and microbial biofilms on the hindgut wall), plays a major role in protection against opportunistic bacteria, fungi and parasites [7, 8, 10]. Honey bee innate immunity is divided into two main categories: humoral and cellular immunity [11]. Humoral immunity involves the production of antimicrobial peptides (AMPs), such as abaecin [12], apidaecin [13], defensin [14] and hymenoptaecin [15], which are released by host cells in response to infection by opportunistic pathogens [10]. Cellular immunity involves processes such as phagocytosis, nodulation and encapsulation, these last two being often accompanied by melanization, a process commonly catalyzed by phenoloxidases that leads to the production of several reactive quinones (e.g., dopachrome) and ultimately melanin, which are very toxic to microbes [16, 17].

Honey bees exposed to antibiotics exhibit dysbiosis and increased susceptibility to opportunistic bacterial pathogens [18, 19]. More recent studies have demonstrated that other anthropogenic chemicals, such as glyphosate, can also perturb the gut microbiota of honey bees [20–24]. Similar cases of dysbiosis have also been observed in other non-target organisms exposed to glyphosate or glyphosate-based formulations [25–30], raising concerns regarding whether glyphosate-induced dysbiosis could affect host immune homeostasis.

Glyphosate is a broad-spectrum herbicide with bacteriostatic properties globally used to destroy unwanted vegetation in crop and non-crop areas and applied at concentrations usually higher than 30 mM (see reference [31] for details regarding glyphosate’s mechanism of action). Besides the effects on the gut microbiota, glyphosate has been also associated with behavioral, developmental and/or neurological changes in honey bee larvae, honey bee adults [32–38] and other non-target organisms [39–41]. Glyphosate can also inhibit melanization in fungi [42] and in the hemolymph of some insects [43].

In this study, we investigated whether glyphosate exposure affects the honey bee immune system. Towards this goal, we performed a series of experiments to investigate the effects of glyphosate on the expression of host immunity-related genes and on the melanization cascade. First, we performed three in vivo experiments in which honey bees were exposed to sublethal concentrations of glyphosate. These experiments were performed in different seasons. In the first two in vivo experiments, we investigated changes in gene expression in gut tissues, whereas in the third in vivo experiment, we extended our assays to whole bee body samples. Second, we conducted ex vivo and in vivo experiments in which bee hemolymph and honey bees, respectively, were exposed to different concentrations of glyphosate to investigate potential consequences for the melanization cascade, an important component of bee immunity.

In all experiments, we included a negative control group (no glyphosate exposure), and an antibiotic-treated comparison group. Antibiotics are known to affect the gut microbiota and the expression of immunity genes in honey bees [18, 19, 44, 45], and this comparison enabled us to verify that our methods were able to detect these shifts. For that comparison group, we used tylosin, an antibiotic commonly used in beekeeping [46] and also known to perturb the gut microbiota of honey bees [21, 22]. Finally, we assayed cultivated bee bacterial pathogens for their susceptility to bee antimicrobial peptides that we found to be affected by glyphosate exposure. Below, we describe these experiments in detail.

## Results

### Effects of glyphosate and tylosin on the transcriptome and microbiome of honey bees

**Fig. 1:**
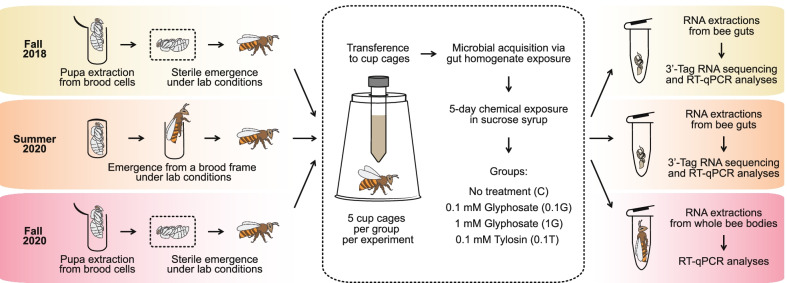
In vivo experiments to investigate the effects of glyphosate and tylosin on the honey bee immune system. Three independent experiments were performed with newly emerged honey bees (*Apis mellifera*) originating from different hives from different seasons. In the fall 2018 and fall 2020 experiments, pupae were extracted from a brood frame and allowed to emerge under sterile conditions, whereas in the summer 2020 experiment, pupae were allowed to emerge naturally from a brood frame kept in the laboratory. In all experiments, healthy newly emerged workers were transferred to cup cages and allowed to acquire their microbiota simultaneously to treatment (0.1 mM glyphosate, 1 mM glyphosate or 0.1 mM tylosin in sucrose syrup) for 5 days. A control group was treated with sucrose syrup only. In the fall 2018 and summer 2020 experiments, RNA was extracted from individual bee guts and pooled for 3′-Tag RNA sequencing (3 pooled guts per sample, 5 samples sequenced per group), whereas in the fall 2020 experiment, RNA was extracted from whole bee bodies (15 individual bees per group) and used in downstream analyses

**Fig. 2:**
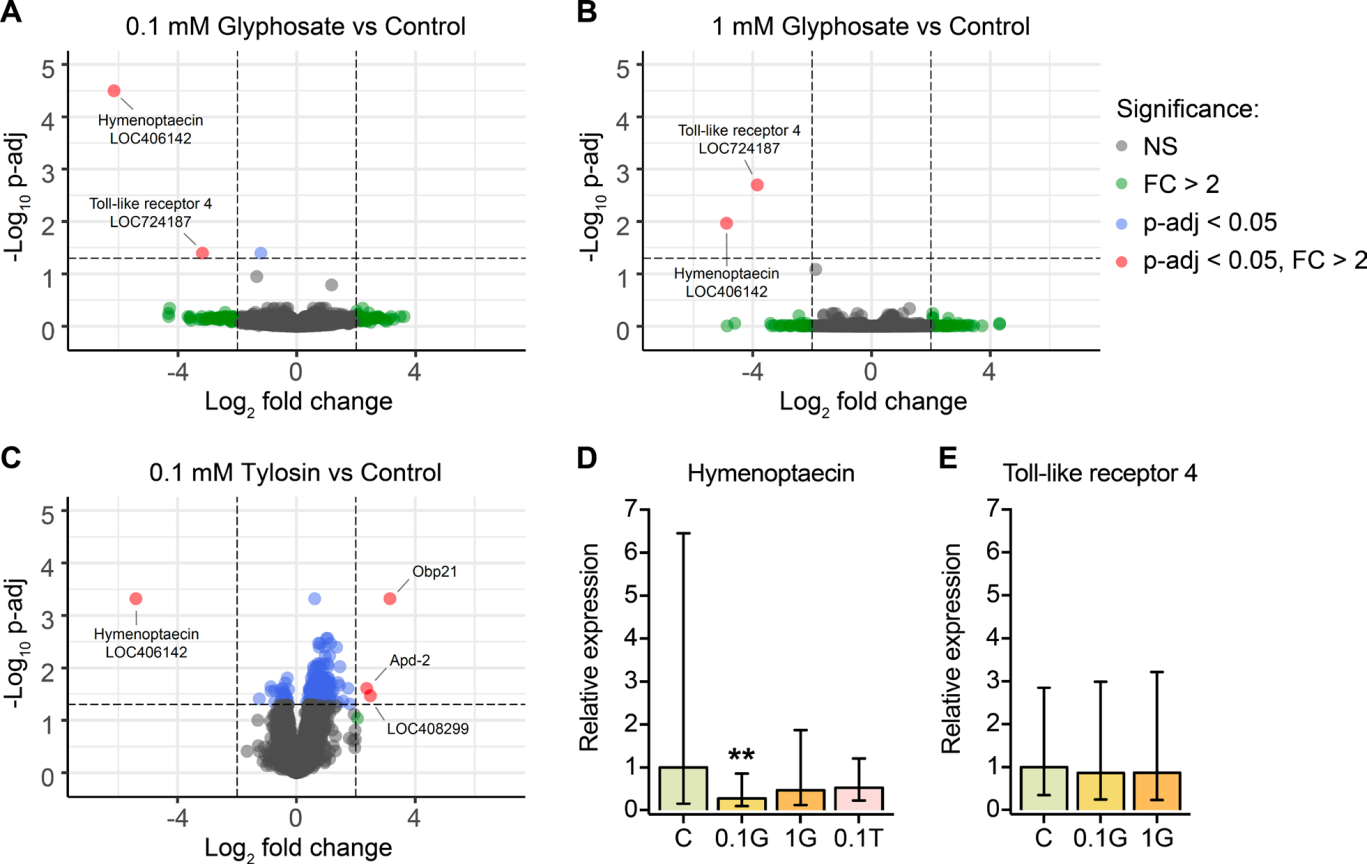
Effects of glyphosate or tylosin exposure on the gut transcriptome of honey bees in fall 2018. **A**–**C** Volcano plots showing differential gene expression in the guts of bees exposed to **A** 0.1 mM glyphosate, **B** 1.0 mM glyphosate or **C** 0.1 mM tylosin, when compared to unexposed, control bees, for a total of 9833 genes. Data points are colored for genes significantly differentially expressed, as follows: blue for *p*-adj < 0.05, green for FC > 2 and red for both *p*-adj < 0.05 and FC > 2; non-significant points are gray. Each group consists of 5 samples, each representative of 3 bee guts. **D**, **E** RT-qPCR expression for the genes **D** hymenoptaecin and **E** Toll-like receptor 4 relative to the housekeeping gene *rps5* in the guts of unexposed and 5 day exposed bees. Each group consists of 15 samples, each representative of a bee gut. Averages and standard deviations are shown as bars and error bars. The linear regression ‘lm’ option in the pcr package in R was applied to estimate differences between control and treatment groups. **p < 0.01

Considering that glyphosate or tylosin exposure affects the gut microbiota of honey bees [20–24], we decided to investigate whether these microbial perturbations could lead to other impacts on honey bee physiology. To address this, we initially investigated changes in the honey bee gut transcriptome due to glyphosate (0.1 or 1 mM) or tylosin (0.1 mM) exposure in experiments performed in fall 2018 and summer 2020 (Fig. 1). For that, RNA was extracted from the guts of 15 bees from each group in each experiment, then pooled in groups of three, giving a total of five pooled samples per group. These samples were submitted for 3′-Tag RNA sequencing, an alternative method for conventional RNA sequencing, which focuses sequencing effort on the 3′ end of mRNAs, reducing sequencing depth per sample, and thus cost [47]. In the fall 2018 experiment, we observed a downregulation for the gene encoding the antimicrobial peptide hymenoptaecin (LOC406142) in the guts of glyphosate- or tylosin-exposed bees when compared to unexposed bees (Fig. 2). The gene encoding a Toll-like receptor 4 (LOC724187) was also downregulated in the guts of glyphosate-exposed bees (Fig. 2A, B). Also, the genes encoding apidermin 2 (Apd-2), an odorant binding protein 21 (Obp21), and a purine nucleoside phosphorylase (LOC408299) were upregulated in the guts of tylosin-exposed bees (Fig. 2C). However, these effects were not observed in the summer 2020 experiment, for which we only detected a few significantly downregulated genes in the guts of 1 mM glyphosate-exposed bees, which encoded a chemosensory protein 1 (CSP1), a phospholipase A2-like (LOC724436), and an unknown protein (LOC100577054) (Additional file 1: Fig. S1). To further investigate the results obtained in the fall 2018 experiment, we used individual RNA samples from control and specific treatment groups as templates for RT-qPCR, giving a total of 15 samples per group, and checked the expression of genes encoding hymenoptaecin (Fig. 2D) and Toll-like receptor 4 (Fig. 2E). Based on the RT-qPCR assays, we found significantly lower expression for hymenoptaecin in gut tissues of the 0.1 mM glyphosate-exposed group, with a 3.6-fold decrease, as well as nonsignificant decreases for both other treatment groups (1 mM glyphosate and 0.1 mM tylosin) (Fig. 2D). However, the RT-qPCR data for the Toll-like receptor 4 gene did not corroborate the findings observed in the 3′-Tag RNA-seq data, and thus we were unable to draw any conclusion regarding the impact of glyphosate on expression of this gene.

**Fig. 3:**
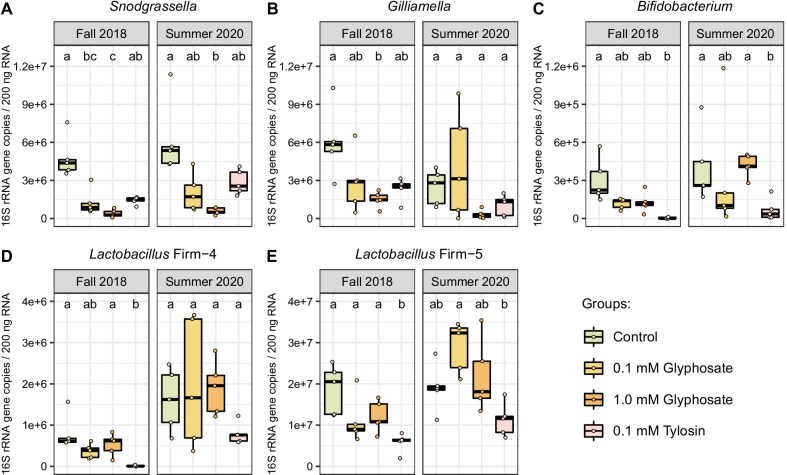
Effects of glyphosate or tylosin exposure on the gut microbiota of honey bees in the fall 2018 and summer 2020 experiments. **A**–**E** Changes in the abundance of 16S rRNA gene transcripts for **A** *Snodgrassella*, **B** *Gilliamella*, **C** *Bifidobacterium*, **D** *Lactobacillus* Firm-4, and **E** *Lactobacillus* Firm-5 in the guts of honey bees exposed to 0.1 mM glyphosate, 1.0 mM glyphosate or 0.1 mM tylosin for 5 days, when compared to unexposed, control bees. Each group consists of 5 samples, each representative of 3 bee guts. Each experiment was analyzed individually, and significance of differences among groups was measured with Kruskal–Wallis tests followed by Dunn’s multiple-comparison tests. Groups with different letters are significantly different at *p* < 0.05

We also investigated perturbations in the gut microbiota caused by glyphosate or tylosin exposure in both experiments (Fig. 3 and Additional file 1: Fig. S2). For the fall 2018 experiment, we found results similar to those of previous studies [22]: significant decreases in *S. alvi* and *Gilliamella* spp. in glyphosate-exposed groups (Fig. 3A, B), and significant decreases in *Bifidobacterium* spp. and *Lactobacillus* Firm-4 and Firm-5 in the tylosin-exposed group (Fig. 3C–E). However, fewer significant changes were observed for the summer 2020 experiment, with decreases in abundance only for *S. alvi* in the 1 mM glyphosate-exposed group, and *Bifidobacterium* spp. in the 0.1 mM tylosin-exposed group (Fig. 3 and Additional file 1: Fig. S2), suggesting that the greater the perturbation of the microbiome the greater the impact on the immune system. However, this is not conclusive, since these experiments were performed in slightly different ways (Fig. 1): experimental bees from the fall 2018 experiment came from late-stage pupae extracted from brood cells, while bees from the summer 2020 experiment were allowed to emerge naturally from brood frames kept in the laboratory.

**Fig. 4:**
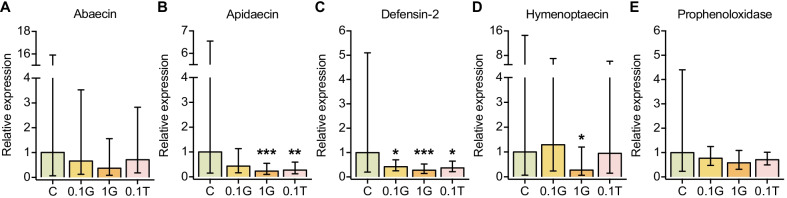
Effects of glyphosate or tylosin exposure on the expression of immunity genes in honey bees in fall 2020. **A**–**E** RT-qPCR expression for the genes **A** abaecin, **B** apidaecin, **C** defensin-2, **D** hymenoptaecin and **E** prophenoloxidase relative to the housekeeping gene *rps5* in the bodies of unexposed and 5 day exposed bees. n = 17, 11, 19, and 13 for control (C), 0.1 mM glyphosate (0.1G), 1.0 mM glyphosate (1G), and 0.1 mM tylosin (0.1T) groups, respectively. Averages and standard deviations are shown as bars and error bars. The linear regression ‘lm’ option in the pcr package in R was applied to estimate differences between control and treatment groups. *p < 0.05, **p < 0.01, ***p < 0.001

To further explore the effects of glyphosate and tylosin on the bee humoral response and in other bee body compartments, we performed a third (fall 2020) experiment, in which we handled the bees similarly to the fall 2018 experiment, but extracted RNA from whole bee bodies, instead of only guts, and used these RNA samples as templates for RT-qPCR analyses (Fig. 1). We checked the expression of genes encoding the AMPs abaecin, apidaecin, defensin and hymenoptaecin, and found significant downregulations for apidaecin (4.4- and 3.7-fold decreases for the 1 mM glyphosate and 0.1 mM tylosin exposed groups, respectively), defensin-2 (2.4-, 3.7- and 2.7-fold decreases for the 0.1 mM glyphosate, 1 mM glyphosate and 0.1 mM tylosin exposed groups, respectively), and hymenoptaecin (3.7-fold decrease for the 1 mM glyphosate exposed group) (Fig. 4). The data collected from these three experiments demonstrate that glyphosate and tylosin can affect the immune system of honey bees, by changing the expression of AMPs, but this effect may vary according to tissue analyzed, experimental conditions or colony status.

A major question is whether changes in immune expression affects pathogen susceptibility and therefore contributes to the protective effect observed for the bee gut microbiota [48]. Thus, we investigated the susceptibility of an opportunistic pathogen of adult bees, *Serratia marcescens,* to apidaecin (isoforms 1a and 1b) and hymenoptaecin by performing minimum inhibitory concentration (MIC) assays. We tested four strains, Db11 [49], Ss1 [50], kz11 and kz19 [51], and none were inhibited by the AMPs (MIC > 50 μg/mL) (Additional file 1: Fig. S3, Additional file 1: Table S2). In contrast, the bee gut symbiont *S. alvi* strain wkB2 was also tested and exhibited susceptibility to apidaecin 1b (MIC = 25 μg/mL) and hymenoptaecin (MIC = 12.5 μg/mL) (Additional file 1: Fig. S3, Additional file 1: Table S2).

### Effects of glyphosate and tylosin on the melanization cascade of honey bees

The main effects observed for the gut transcriptome and RT-qPCR data were associated with perturbations of the bee immune system, and a recently published study showed that glyphosate can inhibit melanization in the hemolymph of some insects [43]. Therefore, we decided to investigate whether glyphosate affects melanization in honey bees.

Initially, we examined the transcript level of the gene encoding prophenoloxidase, which is involved in the melanization immune response [11]. This gene did not show significant expression differences in guts of control and glyphosate-exposed bees in either the fall 2018 or summer 2020 experiments, based on the transcriptome data, nor in whole bee bodies in the fall 2020 experiment, based on the RT-qPCR data (Fig. 4E).

**Fig. 5:**
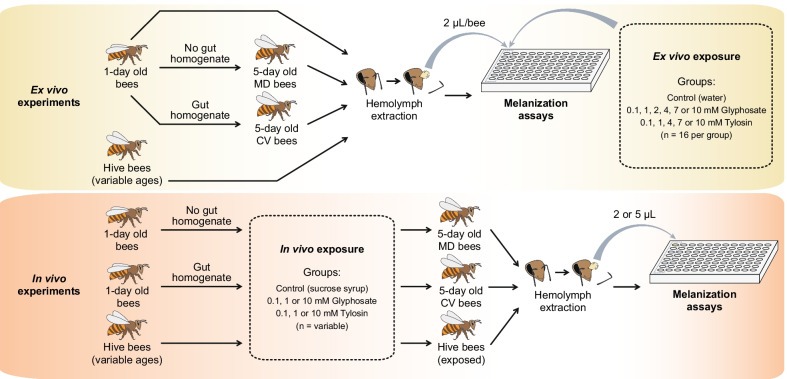
Ex vivo and in vivo experiments to investigate the effects of glyphosate and tylosin on melanization in honey bees. Ex vivo experiments were performed with 1-day old bees, 5-day old bees lacking or containing a normal microbiota, and hive bees. 2 μL of hemolymph were extracted from individual bees and used along with variable concentrations of glyphosate or tylosin (0, 0.1, 1, 2, 4, 7 or 10 mM) in melanization assays. In vivo experiments were performed with 5-day old bees lacking or containing a normal microbiota and hive bees previously exposed to different concentrations of glyphosate or tylosin (0, 0.1, 1 or 10 mM) for 5 days. 2 μL or 5 μL of hemolymph were extracted from exposed bees and used in melanization assays. *MD* microbiota-defective, *CV* conventional microbiota

Then, we investigated the ability of glyphosate (and tylosin for comparison) to inhibit the formation of melanin or intermediates of the melanization cascade, as demonstrated for other insects [43]. We tested a wide range of concentrations (from 0.1 to 10 mM) in two different sets of experiments to make sure we would capture the potential effects of glyphosate (and tylosin) on the bee melanization cascade (Fig. 5).

**Fig. 6:**
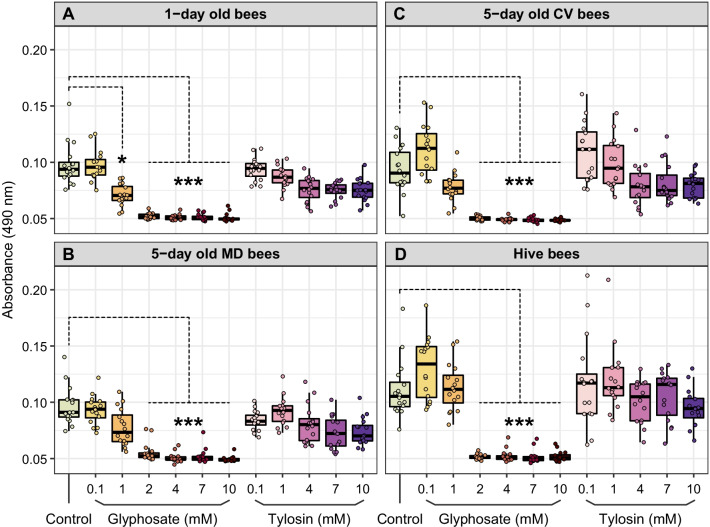
Ex vivo experiments to investigate the effects of glyphosate or tylosin exposure on the melanization cascade in the hemolymph of honey bees. **A**–**D** The formation of dopachrome, an intermediate quinone of the melanization cascade, was measured after adding different concentrations of glyphosate or tylosin to the hemolymph extracted from **A** 1-day old bees, 5-day old bees **B** lacking or **C** containing a normal microbiota, and **D** hive bees. n = 16 samples per group. Samples were incubated for 30 min at 30 °C, and absorbance was measured at 490 nm. Significance between groups in each assay was measured with Kruskal–Wallis tests followed by Dunn’s multiple-comparison tests. *p < 0.05 and ***p < 0.001. *MD* microbiota defective, *CV* conventional microbiota

First, we performed ex vivo experiments to investigate whether glyphosate or tylosin inhibits the formation of an intermediate of the melanization pathway, dopachrome, as well as melanin in the hemolymph of honey bees at different stages of development and microbial acquisition: hemolymph was extracted from 1-day old bees, 5-day old bees lacking or containing a normal microbiota, and also worker bees collected from a hive that were not age-controlled. In all of these scenarios, concentrations of glyphosate higher than 2 mM inhibited the production of dopachrome (Fig. 6) and melanin (Additional file 1: Fig. S4), while tylosin did not inhibit melanization in the honey bee hemolymph. Interestingly, dopachrome formation was also lower in 1 mM glyphosate-exposed hemolymph of 1-day old bees than in unexposed hemolymph (Fig. 6A).

**Fig. 7:**
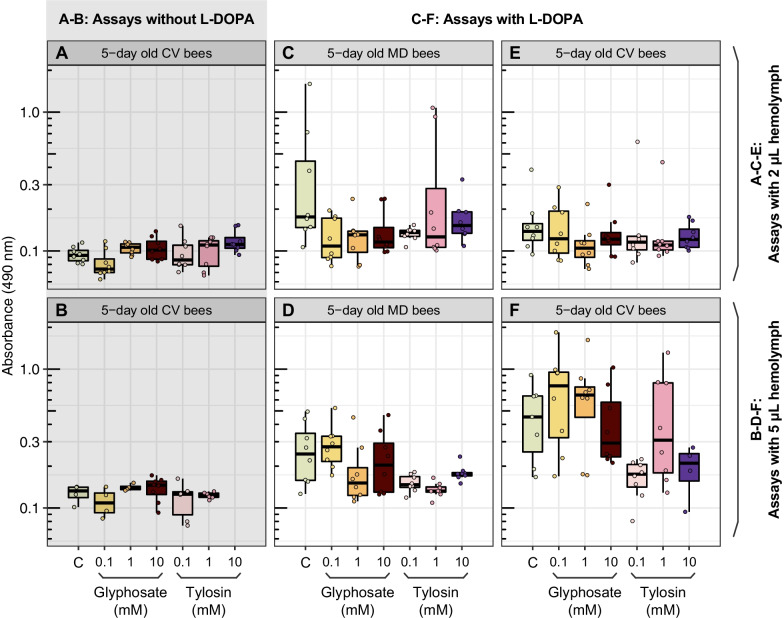
In vivo experiments to investigate the effects of glyphosate or tylosin exposure on the melanization cascade in the hemolymph of honey bees. **A**–**F** Dopachrome formation was measured in the hemolymph of honey bees previously exposed to different concentrations of glyphosate or tylosin. **A**, **B** No l-DOPA was added to the assays. **C**–**F** l-DOPA was added to the assays. Samples were incubated for 30 min at 30 °C, and absorbance was measured at 490 nm. **A**, **C**, **D**, **E** n = 8 samples per group. **B** n = 4 samples for control, 0.1 mM glyphosate and 1 mM glyphosate groups; n = 8 samples for 10 mM glyphosate group, n = 6 samples for 0.1 mM tylosin and 1 mM tylosin groups. **F** n = 8 samples per group, except n = 4 for 10 mM tylosin group. No statistically significant differences were observed between groups in any assay (*p* > 0.05, Kruskal–Wallis tests). *MD* microbiota defective, *CV* conventional microbiota

Unlike the ex vivo experiments, in which we extracted hemolymph from unexposed bees to perform the assays, we performed in vivo experiments, in which we first exposed honey bees to glyphosate or tylosin for 5 days (Additional file 1: Fig. S5), then extracted hemolymph and performed similar assays. This time, we tested whether the chemical could reach the hemolymph after ingestion and cause similar effects, since this represents a more realistic scenario. However, we did not find significant changes in dopachrome or melanin formation between unexposed and exposed bees (Fig. 7A, B), even when adding an exogenous substrate, l-DOPA, to increase the production of melanin and its intermediates (Fig. 7C–F). This was done for 5-day old bees lacking or containing a normal microbiota and older worker bees. The lack of an effect may be because glyphosate, once consumed by the bees, does not accumulate in the hemolymph, instead going to different compartments of the bee body or being taken up by the gut microbiota.

## Discussion

### Glyphosate, similar to antibiotics, can downregulate the expression of AMPs

One of the striking effects of glyphosate on honey bees is its impact on the gut microbiota, drastically reducing the abundance of beneficial bacterial symbionts, such as *S. alvi*. As shown in previous studies, *S. alvi*-monocolonized bees exhibit upregulation of genes encoding apidaecin in the guts [7], as well as abaecin and hymenoptaecin in the abdomens [8] when compared to microbiota-free bees; administration of heat-killed *S. alvi* cells also increases the expression of these AMPs, but in a less controlled way [8]. In our study, control bees were colonized with a normal microbiota and, therefore, were probably expressing basal levels of AMPs. Under these conditions, glyphosate exposure, besides killing *S. alvi* cells, promoted downregulation of the genes encoding apidaecin and defensin in whole bee body samples, as well as hymenoptaecin in both bee gut and whole bee body samples. These effects were observed in two out of three trials and, therefore, may vary according to tissue analyzed, experimental conditions or colony status, as suggested in other bee studies [32, 52].

Tylosin, an antibiotic commonly used in beekeeping, also affected the bee gut microbiota, as in previous studies [21, 22], and reduced the expression of apidaecin and defensin-2 in whole bee body samples, similar to glyphosate in this study and other antibiotics [19]. The effects of tylosin on the honey bee immune system is not a surprise, as previous studies have demonstrated negative effects of antibiotics on the bee immune system [19] and resistance to opportunistic pathogens [45]. In the transcriptome analysis of the fall 2018 experiment, several genes were expressed differently between tylosin-exposed and control groups (*p*-adj < 0.05), but mostly with fold-changes less than two; this was not observed in the summer 2020 experiment. Thus, the effects of these agrochemicals on expression of immunity genes may depend on colony status.

It remains to be elucidated whether the observed downregulation of AMPs is a direct effect of glyphosate or tylosin on the bee immune system or an indirect effect due to perturbations on the gut microbiota. AMP expression is typically modulated by exposure to specific opportunistic microbes, such as Gram negative and Gram positive bacteria, fungi, and microsporidia [10, 12, 13, 15, 53, 54]. Some studies have also suggested that AMPs may be involved in the maintenance of microbiome homoeostasis [55]. In honey bees, the production of AMPs in the gut may play a dual role by inhibiting the proliferation of opportunistic microbes and by regulating the proliferation of the native bacteria. For example, while *S. alvi* strains appear to be tolerant of apidaecin, they are less tolerant of hymenoptaecin [7].

In insects, AMP expression is controlled by one of the two major immune response pathways, the immune deficiency pathway (Imd) or the Toll pathway [56]. Downregulation of genes encoding components of these pathways, such as receptors, transcriptional factors, or AMPs may favor the proliferation of opportunistic microbes, which wouldn’t grow otherwise [57]. In some instances, pathogens may find mechanisms to evade the host natural defenses; for example, *S. marcescens*, an opportunistic pathogen of worker bees, does not stimulate the host expression of AMPs [51] and is not susceptible to the AMPs apidaecin and hymenoptaecin, at least under in vitro conditions (Additional file 1: Fig. S3). Nonetheless, *S. marcescens* can take advantage of microbiome perturbations caused by exposure to anthropogenic chemicals, such as antibiotics and glyphosate, and cause disease [18, 21, 23].

As caveats, in this study we primarily examined transcriptomic changes in guts and AMP expression in whole bee bodies of young worker bees, and therefore cannot assess effects of glyphosate or tylosin on expression of other genes in other specific tissues or in older worker bees, such as foragers. Other studies have shown differential expression of AMPs according to body part and age. For example, the AMP defensin is mainly expressed in the head and thorax of forager bees [58]. Also, the transcriptomes of foragers are affected by glyphosate-based formulations, but this exposure seems to increase rather than decrease apidaecin expression [38]. Moreover, other studies have demonstrated that honey bee larvae exposed to glyphosate exhibit variable transcriptional changes [32, 52].

### Does glyphosate or tylosin inhibit melanization in honey bees?

Another major component of the immune system in insects and many other invertebrates is the melanization of pathogens and injured tissues [59]. The melanization process involves a series of redox reactions, typically mediated by phenoloxidases, which once activated upon wound or infection, oxidize phenols in the hemolymph into toxic quinones, which polymerize nonenzymatically and form melanin [60]. Then, melanin and its highly reactive and toxic precursors surround and expose the invader to reactive oxygen species culminating in its death [61].

Previous studies have demonstrated that glyphosate inhibits melanin production in the fungus *Cryptococcus neoformans* [42] and in the hemolymph of the insects *Galleria mellonella* and *Anopheles gambiae* [43]. Glyphosate does not directly inhibit phenoloxidases, but instead affects the melanization cascade by acting as a synergistic antioxidant and disrupting the redox reactions required for melanization, which halts the production of melanin [43]. In this study, we saw that concentrations of glyphosate higher than 2 mM inhibit melanization in the hemolymph of honey bees in ex vivo studies, but we were unable to recapitulate these findings in in vivo studies. Once bees are exposed to glyphosate, this herbicide may pass through the hemolymph to reach different compartments of the bee body, but may not accumulate in the hemolymph. Based on previous studies on glyphosate-induced perturbations of the bee gut microbiota, it is expected that a portion of the ingested glyphosate is not absorbed, passes intact into the hindgut, and reaches high enough concentrations to perturb the microbiota at least in the ileum, the proximal compartment of the hindgut, where *S. alvi* resides. *S. alvi* abundance is drastically reduced upon glyphosate exposure, based on results of several studies [21–24].

Interestingly, *F. perrara*, another gut symbiont commonly found in honey bees, upregulates the expression of genes associated with the melanization cascade—as well as AMP production—forming a scab in the pylorus, region connecting the midgut to the ileum [9]. However, glyphosate seems not to affect *F. perrara* abundance in the bee gut, although this bacterium encodes the susceptible version of the enzyme that glyphosate inhibits [21]. It is unclear whether melanization inhibition could affect *F. perrara* colonization patterns in the bee gut, with potential consequences for host health.

Unlike glyphosate, tylosin exposure does not affect melanization in the bee hemolymph, at least under the tested conditions. Tetracycline, another antibiotic commonly used in beekeeping [46], does not affect melanin content or tyrosinase activity in human melanocytes [62], suggesting that the same may occur in bee hemocytes. However, the lack of effects on melanization does not hold true for all antibiotics, as demonstrated for fluoroquinolone antibiotics, such as ciprofloxacin, norfloxacin and moxifloxacin, which decrease melanin content and tyrosinase activity in human melanocytes [63, 64].

In summary, our results show that glyphosate, unlike tylosin, can inhibit melanization in the bee hemolymph, but this would only happen in vivo if bees are exposed to high levels, such that the herbicide reaches concentrations of at least 2 mM in the hemolymph. This could happen, for example, if forager bees are directly exposed to herbicide formulations when foraging.

## Conclusions

This study provides experimental evidence that glyphosate exposure, similar to antibiotics such as tylosin, not only can cause dysbiosis in the honey bee *Apis mellifera*, as shown previously [21–24], but also can alter immune response pathways, by downregulating the expression of host-produced AMPs. We also observed that glyphosate can potentially affect melanization in the bee hemolymph, depending on level of exposure and delivery to the hemolymph. Since AMP production and melanization are two major components of the innate immune system of honey bees, disruption of these pathways may promote negative consequences to host health, such as increased susceptibility to infection and reduced lifespan.

## Methods

### In vivo experiments for transcriptome studies

To investigate the effects of glyphosate exposure on the expression of AMPs and other immunity-related genes in gut tissues or in whole bee bodies, we performed three in vivo experiments with newly emerged honey bees (*Apis mellifera*) originating from different hives kept at the University of Texas at Austin (UT-Austin). As we describe below, these experiments were performed in different seasons (fall 2018, summer 2020 and fall 2020), and the experimental conditions slightly varied based on the findings we obtained in past experiments (Fig. 1).

In the fall 2018 experiment, late-stage pupae (with eyes pigmented but lacking movement) were extracted from a brood frame, transferred to clean plastic bins and placed in an incubator at 35 °C and ~ 60% relative humidity to simulate hive conditions until emerging as adults. Healthy newly emerged workers (NEWs) were transferred to cup cages containing sterile sucrose syrup and bee bread mixed with a gut homogenate so they could acquire their native microbiota [22]. Cup cages were divided into a control group, which was fed sterile sucrose syrup, and three treatment groups, which were fed 0.1 mM glyphosate in sterile sucrose syrup, 1 mM glyphosate in sterile sucrose syrup or 0.1 mM tylosin in sterile sucrose syrup, respectively, for 5 days. The glyphosate concentrations were chosen to be in the range detected in nectar and pollen of recently sprayed plants in a semi-field experiment [65]. Tylosin is an antibiotic commonly used in beekeeping and served in this study as a comparison treatment, expected to have a major effect, as antibiotics including tylosin are known to disrupt the gut microbiota of honey bees [44, 45] and to affect the expression of immunity-related genes [19]. The tylosin concentration used is far below that recommended for hive applications.

Each group consisted of 4 cup cages, with 26–30 bees per cup cage. In the end of the treatment, 15 bees were sampled from each group, placed in 5 mL Falcon tubes, and stored at − 80 °C until further analyses. As described below, RNA was extracted from individual bee guts and pooled for 3′-Tag RNA sequencing (3 RNA samples per pooled sample, 5 pooled samples per group) or checked individually for RT-qPCR analysis (15 RNA samples per group). Samples from this experiment were produced as part of a recently published study in which we investigated the effects of glyphosate on the honey bee gut microbiota [22].

The summer 2020 experiment was performed to replicate the fall 2018 experiment with some altered conditions. In 2020, pupae were allowed to emerge naturally from a brood frame kept in an incubator at 35 °C and ~ 60% relative humidity. This method enables a more natural emergence process, wherein bees are exposed to environmental microbes present on the frame before the experimental exposure to the native microbiota in gut homogenates. In the hive, bees would also be exposed to environmental microbes and would acquire their native microbiota by interaction with nurse bees or fecal material. One-day-old bees were transferred to cup cages and treated as described for the fall 2018 experiment.

The fall 2020 experiment was performed similarly to the fall 2018 experiment, but with bees from a different hive. This time, RNA was extracted from whole bee bodies to extend the findings from bee guts to other bee body compartments.

### Dissections and RNA extractions

For the fall 2018 and summer 2020 experiments, bee guts were dissected with flame-sterilized forceps under aseptic conditions and on ice, and RNA was extracted from individual guts using the Quick-RNA^™^ Miniprep kit (Zymo Research^®^). For the fall 2020 experiment, total RNA was extracted from whole bee bodies. Guts (fall 2018, summer 2020) or whole bee bodies (fall 2020) were crushed in 100 μL of RNA Lysis Buffer, resuspended in a total of 600 μL of the same solution, and transferred to a capped vial containing ~ 0.5 mL of 0.1-mm Zirconia beads (BioSpec Products Inc.). Samples were bead-beaten for 2 × 30 s, centrifuged at 14,000 rpm for 30 s, and transferred to a new microtube. After this step, extraction followed the protocol provided by Zymo Research®. Final RNA samples were eluted in 50 μL of water and stored at − 80 °C.

### Library preparation for 3′-Tag RNA sequencing

Aliquots of RNA samples from the fall 2018 and summer 2020 experiments were initially pooled according to cup cage source (1000 ηg of each RNA sample), giving a final number of 40 pooled RNA samples, 20 samples per experiment, 5 samples per group, 3 bees per sample. Then, pooled samples were diluted to a final RNA concentration of 100 ηg/mL and submitted for 3′-Tag RNA sequencing (Admera Health Inc.). QuantSeq 3′ mRNA-Seq Library Prep Kit FWD for Illumina (Lexogen Inc.) was used to create libraries for 1 × 50 bp single-end sequencing on an Illumina HiSeq X instrument (Genohub project # 3979681), which generated a total of 473,838,012 reads, ranging from 9,772,385 to 13,966,825 reads per library.

### Processing of 3′-Tag RNA sequencing data

3′-Tag RNA-seq data were processed following the scripts provided by Lexogen Inc. at https://www.lexogen.com/quantseq-data-analysis. Sequence visualization and quality control were performed with FastQC [66]. Adapter contamination, polyA tail read through, and low quality tails were trimmed using the bbduk.sh script in the BBMap package [67]. Then, the STAR aligner [68] was used to build a STAR index using the most updated versions of the *Apis mellifera* genome (GCF_003254395.2_Amel_HAv3.1_genomic.fna) and gene annotations (GCF_003254395.2_Amel_HAv3.1_genomic.gtf), and used to align and map the reads to the *Apis mellifera* genome, generating gene counts files which were used in downstream analyses. Differential gene expression analysis was performed using DESeq2 [69] in R version 3.5.2 [70]. Gene counts were normalized, and low counts were filtered whenever there were less than 5 samples with normalized counts greater than or equal to 5, giving a final number of 9833 genes. The comparisons of gene expression were made between the control and each treatment in each experiment, and a gene was considered significant if the false discovery rate (FDR) was less than 0.05 and the absolute fold change more than 2.

### Gene relative expression analyses

We performed RT-qPCR analyses to confirm the findings observed in the 3′-Tag RNA sequencing data for the fall 2018 experiment, and to investigate potential changes in the expression of specific immunity-related genes in the fall 2020 experiment. For this, cDNA was synthesized from 800 ηg of each individual RNA sample from each of these two experiments using the qScript cDNA Synthesis Kit (QuantaBio, USA). cDNA samples (120 in total) were ten-fold diluted to be used as template for RT-qPCR analyses. The fold-change in expression between control and treated bees were determined for the genes encoding hymenoptaecin and Toll-like receptor 4 for the fall 2018 experiment, and abaecin, apidaecin, defensin, hymenoptaecin and prophenoloxidase for the fall 2020 experiment. For these measures, 10 μL reactions were carried out on 384-well plates on a Thermo Fisher ViiA7 instrument using 5 μL of iTaq Universal SYBR Green Supermix (Bio-Rad Inc.), 0.05 μL of each forward and reverse 100 μM primer (Additional file 1: Table S1), 3.9 μL of H_2_O, and 1.0 μL of template cDNA. The cycling conditions consisted of an initial cycle of 50 °C for 2 min and 95 °C for 2 min, followed by 40 cycles of a two-step PCR of 95 °C for 15 s and 60 °C for 1 min. Expression levels were measured in triplicate for each biological replicate and normalized against the housekeeping gene *rps5* (Additional file 1: Table S1) [71]. Relative expression was performed by means of the ΔΔC_T_ method, and differences in expression between control and treatment groups were investigated using the linear regression ‘lm’ test in the pcr package [72] in R version 3.5.2 [70]. *p* values lower than 0.05 were considered statistically significant. No RT-qPCR analyses were performed for RNA samples from the summer 2020 experiment since we did not observe any significant changes in the 3′-Tag RNA sequencing data.

### Microbial abundance and composition analyses

For the fall 2018 and summer 2020 experiments, cDNA was synthesized from 2 μL of each normalized RNA sample that was also used for the 3′-Tag RNA-seq library preparation. cDNA samples (40 in total) were ten-fold diluted to be used as templates for 16S rRNA library preparation and qPCR analyses.

16S rRNA library preparation consisted of two PCR reactions performed as described in [22]. Briefly, PCR 1 amplified the V4 region of the 16S rRNA gene and was performed in 20 μL triplicate reactions containing 0.5 μL of forward (8 μM 515F) and reverse (8 μM 806R) primers (Additional file 1: Table S1), 8 μL of 2.5× 5PRIME HotMasterMix (Quantabio, USA) and 1 μL of template cDNA. Cycling conditions consisted of 94 °C for 3 min; 30 cycles of 94 °C for 45 s, 50 °C for 60 s, 72 °C for 90 s, then 72 °C for 10 min. PCR 2 attached dual indices and Illumina sequencing adapter to the products of PCR 1 and consisted of 25 μL single reactions containing a unique combination of 2 μL of 5 μM index primers (see Additional file 1: Table S1), 10 μL of 2.5× 5PRIME HotMasterMix (Quantabio, USA) and 5 μL of PCR 1 product. Cycling conditions consisted of 94 °C for 3 min; 10 cycles of 94 °C for 20 s, 55 °C for 15 s, 72 °C for 60 s; then 72 °C for 10 min. For both PCR reactions, products were purified with 0.8× HighPrep™ PCR magnetic beads (MagBio, USA) and quantified fluorometrically (Qubit, Thermo Fisher Scientific Inc.). 50 ηg of each sample was pooled, and the resulting library was diluted to a final concentration of 50 ρM. The diluted library was loaded onto an Illumina iSeq cartridge according to the manufacturer’s instructions and subjected to Illumina sequencing on the iSeq platform (2 × 150 sequencing run, instrument model number: FS10000184). 5% PhiX was used to increase library diversity.

Illumina sequence reads were demultiplexed according to their barcode sequences by the iSeq software and processed in QIIME 2 version 2019.10 [73]. Downstream analyses were performed with forward reads, because of insufficient overlap between forward and reverse reads. Primer sequences were removed using the cutadapt plugin [74] and the reads were truncated to length of 120 bp. Trimmed reads were filtered and denoised, and chimeric reads were removed using the DADA2 plugin [75]. Taxonomy was assigned to amplicon sequence variants (ASVs) using the SILVA database in the feature-classifier plugin [76]. Reads with lower than 0.1% abundance were removed using the feature-table plugin, as well as unassigned, mitochondrial, and chloroplast reads using the taxa filter-table plugin.

qPCR analyses were performed as described in the previous section using universal bacterial 16S rRNA gene primers (Additional file 1: Table S1). Total bacterial 16S rRNA gene transcripts were estimated by standard curves from amplification of the cloned target sequence in a pGEM-T vector (Promega). For specific bacterial species, 16S rRNA gene transcripts were estimated by multiplying the percent relative abundance of each species (obtained by 16S rRNA amplicon sequencing) by the total bacterial 16S rRNA gene transcripts (obtained by qPCR).

### Minimum inhibitory concentration assays

Four strains of *Serratia marcescens* (Db11, Ss1, kz11 and kz19) were cultivated in the presence of different concentrations of the AMPs apidaecin 1a, apidaecin 1b and hymenoptaecin (synthesized by NovoPro Bioscience Inc.) to determine the minimum inhibitory concentration (MIC). *Escherichia coli* strain K12 and *S. alvi* strain wkB2 were tested for comparisons. Assays were performed as described in [7]. Briefly, solutions of 100 μg/mL of each AMP were serially diluted two-fold in 50% brain heart infusion broth (BHI) in 96-well plates, leaving a volume of 100 μL in each well. Bacterial strains were cultured on heart infusion agar with 5% sheep blood at 35 °C and 5% CO_2_. Colonies from overnight cultures were first diluted in 50% BHI to obtain an optical density (OD) of 0.5 at 600 nm, then 100-fold diluted in 50% BHI. Finally, 100 μL of diluted cultures were transferred to the wells. Final concentrations of AMPs in the wells ranged from 0.78 to 50 μg/mL. Plates were incubated at 35 °C and 5% CO_2_ and OD was measured at 600 nm every 24 h for 48 h. MIC was defined as the lowest concentration of AMP that inhibited growth of bacteria compared to controls.

### Ex vivo and in vivo experiments for melanization assays

During winter 2020, we performed ex vivo and in vivo experiments with honey bee hemolymph and honey bees, respectively, to examine the effects of glyphosate on melanization, which is a key part of the immune response. For the ex vivo experiments, a brood frame and hive bees (not age-controlled) were collected from a hive on the UT-Austin campus and transferred to an incubator. The next day, 2 μL of hemolymph were extracted from newly-emerged bees and worker bees as described in [77], transferred to 96-well plates on ice, then preserved at − 80 °C. Other newly emerged bees were transferred to cup cages and split into two groups, which were provided sterile sucrose syrup and bee bread. Only one of these groups was allowed to acquire a normal microbiota by adding to the bee bread a gut homogenate suspension, as described in [22]. Then, 2 μL of hemolymph were also extracted from 5-day old microbiota-defective bees and 5-day old bees with a conventional microbiota. These bees were not exposed to glyphosate or tylosin. For the melanization assays, plates were thawed on ice, added 140 μL of distilled water, 20 μL of a phosphate-buffered saline (PBS) solution (150 mM NaCl, 10 mM Na_2_HPO_4_, pH 6.5) and 18 μL of glyphosate or tylosin solution to give a final concentration in the range of 0.1 to 10 mM. For measurements of dopachrome formation, an intermediate of the melanization pathway, plates were incubated at 30 °C for 30 min and absorbances were measured at 490 nm. After that, plates were incubated at room temperature for 5 days after which absorbances were measured at 490 nm again to determine melanin production.

For the in vivo experiments, newly emerged bees and hive bees (not age-controlled) were treated with different concentrations of glyphosate or tylosin (0.1, 1 or 10 mM) for 5 days. Before the beginning of the chemical treatment, newly emerged bees were split into two main groups, in which only one was allowed to acquire the normal microbiota by providing gut homogenates to the bee bread, as described in [22]. Then, 2 μL of hemolymph were extracted from control and treatment bees, transferred to 96-well plates on ice, and frozen at − 80 °C. For the melanization assays, only 140 μL of distilled water and 20 μL of a PBS solution (150 mM NaCl, 10 mM Na_2_HPO_4_, pH 6.5) were added to the plates. No chemical solution was added this time, since bees were previously exposed to glyphosate or tylosin. In some plates, 20 μL of a 6 mg/mL l-DOPA solution was added to speed up the melanization reaction. Plates were incubated at 30 °C for 30 min and absorbances were measured at 490 nm. To confirm the results from the second set of in vivo melanization experiments, we repeated the assays with 5 μL of hemolymph, as we considered that a higher concentration of hemolymph could favor the melanization reaction.

### Chemicals and solutions

Glyphosate standard was purchased from Research Products International, USA (Lot: 32612–38399). Tylosin tartrate was purchased from GoldBio, USA (Lot: 2313.081915A). 3-(3,4-Dihydroxyphenyl)-l-alanine (l-DOPA) was purchased from TCI Chemicals, USA (Lot: UF6JK-JD). For in vitro experiments, glyphosate, tylosin and l-DOPA were dissolved in distilled water. For in vivo experiments, glyphosate and tylosin were initially dissolved in distilled water, then diluted to the final concentration with filter-sterilized 0.5 M sucrose syrup.

## Supplementary Information

**Additional file 1.** Supplementary figures and tables.

**Additional file 2.** Supplementary dataset.

## Data Availability

Sequencing data are available on NCBI BioProject PRJNA703064. Other data are included in this published article and its Additional information files (Additional file 1 and Additional file 2).

## Abbreviations

AMP: Antimicrobial peptide
BHI: Brain heart infusion
cDNA: Complementary deoxyribonucleic acid
l-DOPA: 3-(3,4-Dihydroxyphenyl)-l-alanine
MIC: Minimum inhibitory concentration
NCBI: National Center for Biotechnology Information
OD: Optical density
PBS: Phosphate-buffered saline
RNA: Ribonucleic acid
rRNA: Ribosomal RNA
RT-qPCR: Reverse transcription quantitative polymerase chain reaction
