# Supplementary material for: Glyphosate induces immune dysregulation in honey bees
Source: Anim Microbiome. 2022 Feb 22;4:16. doi: 10.1186/s42523-022-00165-0 (PMC8862317; doi:10.1186/s42523-022-00165-0)
Supplement: Supplementary file 1 — Additional file 1. Supplementary figures and tables. [file 42523_2022_165_MOESM1_ESM.pdf]

Supplementary Material

Glyphosate induces immune dysregulation in honey bees

Erick V. S. Motta*, J. Elijah Powell, Nancy A. Moran*

Affiliation:
Department of Integrative Biology, University of Texas at Austin, Texas, USA

*Corresponding authors:
Erick V. S. Motta and Nancy A. Moran
2506 Speedway, Austin, TX 78712
Emails: erickvsm@utexas.edu and nancy.moran@austin.utexas.edu

This PDF file includes:

- Supplementary Figures S1 to S5
- Supplementary Tables S1 to S2
- Supplementary References

Supplementary Figures

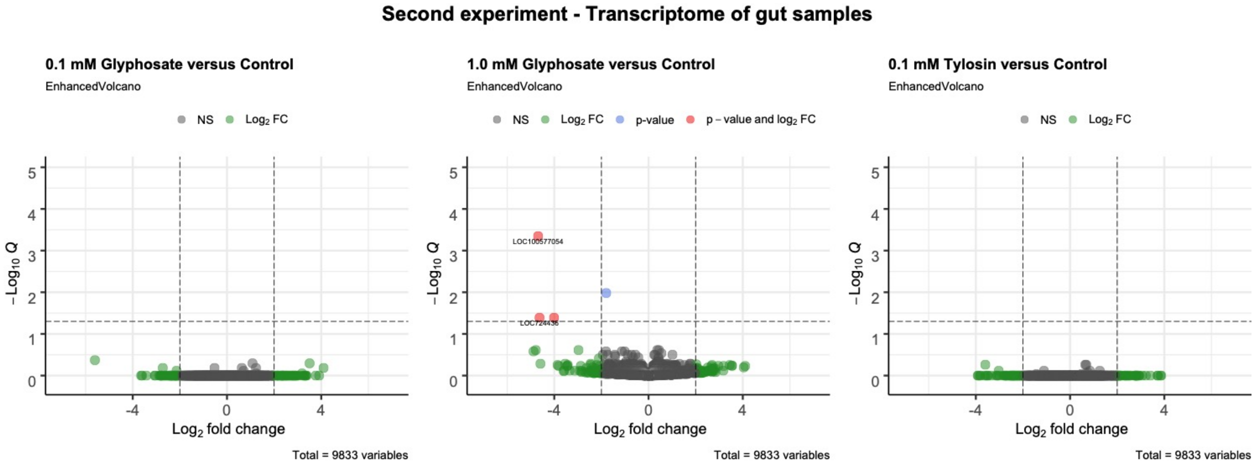

Figure S1. Effects of glyphosate or tylosin exposure on the transcriptome of 5-day old honey bees in the summer 2020 experiment. Volcano plots showing differential gene expression in the guts of bees exposed to 0.1 mM glyphosate, 1.0 mM glyphosate or 0.1 mM tylosin, when compared to unexposed, control bees, for a total of 9833 genes. Data points are colored for genes significantly differentially expressed, as follows: blue for p-adj < 0.05, green for FC > 2 and red for both p-adj < 0.05 and FC > 2; non-significant points are gray. Each group consists of 5 samples, each representative of 3 bee guts.

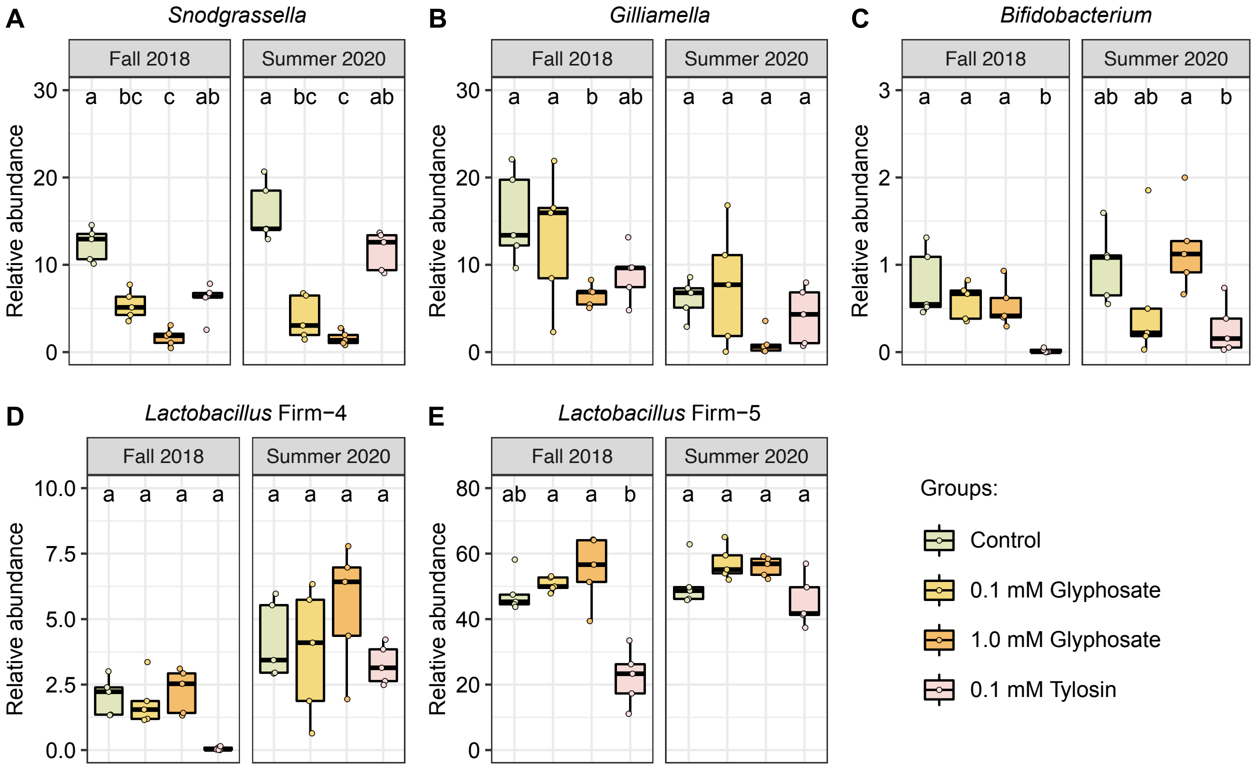

Figure S2. Effects of glyphosate or tylosin exposure on the gut microbiota of honey bees in the fall 2018 and the summer 2020 experiments. (A-E) Changes in the relative abundance of 16S rRNA gene transcripts for **(A)** *Snodgrassella*, **(B)** *Gilliamella*, **(C)** *Bifidobacterium*, **(D)** *Lactobacillus* Firm-4, and **(E)** *Lactobacillus* Firm-5 in the guts of honey bees exposed to 0.1 mM glyphosate, 1.0 mM glyphosate or 0.1 mM tylosin for 5 days, when compared to unexposed, control bees. Each group consists of 5 samples, each representative of 3 bee guts. Significance between groups was measured with Kruskal-Wallis tests followed by Dunn's multiple-comparison tests. Groups with different letters are significantly different at $p < 0.05$.

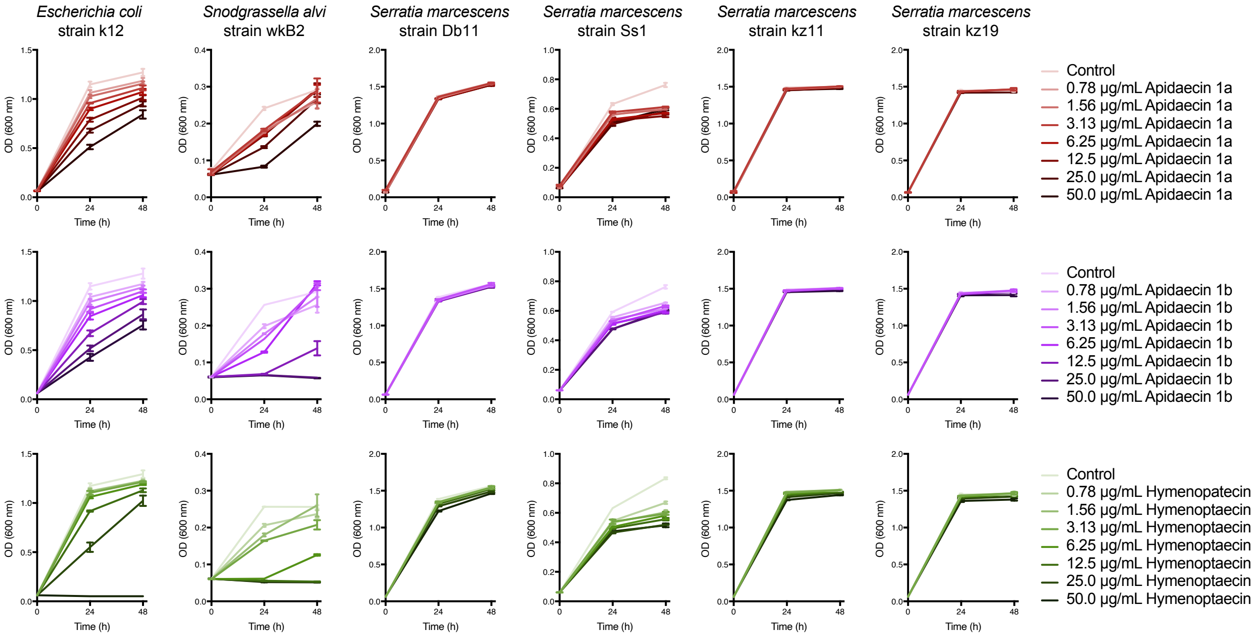

Figure S3. Bacterial growth curves for the minimum inhibitory concentration (MIC) assays for the antimicrobial peptides apidaecin 1a (top), apidaecin 1b (middle) and hymenoptaecin (bottom).

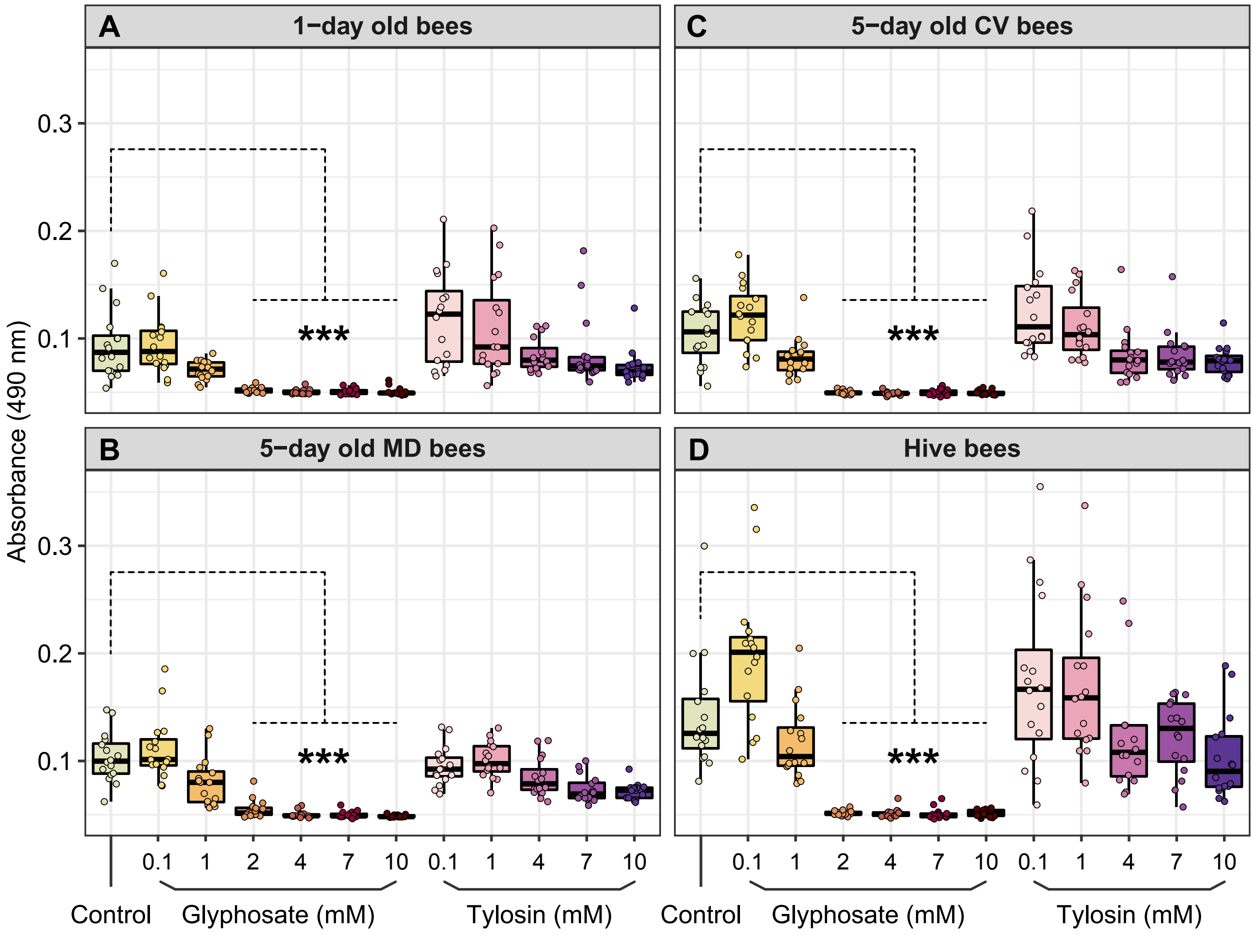

Figure S4. Continuation of the *ex vivo* experiments presented in Figure 3. After measuring dopachrome formation, samples were incubated at room temperature for 5 days after which melanin formation was measured by checking absorbance at 490 nm. n = 16 samples per group. Significance between groups was measured with Kruskal-Wallis tests followed by Dunn's multiple-comparison tests. *** $p < 0.001$. MD = microbiota defective. CV = conventional microbiota.

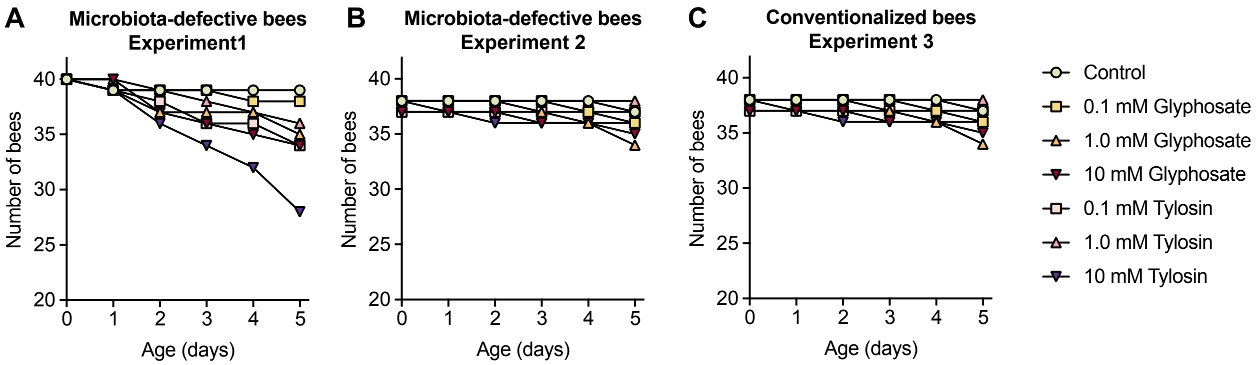

Figure S5. Initial exposure of honey bees to different concentrations of glyphosate or tylosin for subsequent extraction of hemolymph. One-day old honey bees were split into groups to be treated with glyphosate (0.1, 1 or 10 mM) or tylosin (0.1, 1 or 10 mM). **(A)** In the first experiment, bees were not allowed to acquire the normal microbiota (microbiota-defective bees). **(B)** In the second and **(C)** third experiments, bees were allowed to acquire the normal microbiota by providing gut homogenates to the bee bread (conventionalized bees). Hemolymph was extracted from contro and treatment bees to perform melanization assays presented in Figure 5.

Supplementary Tables

Table S1. Primer sets used for PCR assays.

Purpose [Reference]	Forward (5'-3')		Reverse (5'-3')	
Abaecin [1]	Aba-F	ATCTTCGCACTACTCGCCAC	Aba-R	TCGGATTGAATGGTCCCTGA
Apidaecin [2]	Api-F	TTTTGCCTTAGCAATTCTTGTTG	Api-R	GTAGGTCGAGTAGGCGGATCT
Defensin-2 [3]	Def-2F	GGGTAACGTGCGACGTTTTA	Def2-R	GCAACTACCGCCTTTACGTC
Hymenoptaecin [this study]	Hym-F	CGCCTATGGTGGACTAAACA	Hym-R	CAGGACCACGTTGAACTTCA
Prophenoloxidase [this study]	Ppo-F	TAGATTCCCTTGTTGCCAGT	Ppo-R	CGATCACGCCATCTTTCTAG
Toll-like receptor 4 [this study]	Toll-F	GTGAATGATGCGAAGATTTCCA	Toll-R	TTACAGTGCCGAATTGAGGG
5S ribosomal RNA (*rps5*) [3]	Rps5-F	AATTATTTGGTCGCTGGAATTG	Rps5-R	TAACGTCCAGCAGAATGTGGTA
16S ribosomal RNA [3]	27F	AGAGTTTGATCCTGGCTCAG	355R	CTGCTGCCTCCCGTAGGAGT
16S V4 PCR 1 [4]	Hyb515F	TCGTCGGCAGCGTCAGATGTGTA TAAGAGACAGGTGYCAGCMGCC GCGGTA	Hyb806R	GTCTCGTGGGCTCGGAGATGTGT ATAAGAGACAGGGACTACHVGGG TWTCTAAT
Index primers [Nextera PCR 2]	Hyb_Fnn_i5	AATGATACGGCGACCACCGAGAT CTACAC NNNNNNNN TCGTCGGC AGCGTC	Hyb_Rnn_i7	CAAGCAGAAGACGGCATACGAGA T NNNNNNNN GTCTCGTGGGCTC GG

Table S2. Minimum inhibitory concentrations (MICs) of antimicrobial peptides to bee gut bacteria and comparison bacterial taxa.

Bacteria	Strain	MIC (µg/mL)		
		Apidaecin 1a	Apidaecin 1b	Hymenoptaecin
Snodgrassella alvi	wkB2	>50	25	12.5
Serratia marcescens	Db11	>50	>50	>50
	Ss1	>50	>50	>50
	kz11	>50	>50	>50
	kz19	>50	>50	>50
Escherichia coli	K12	>50	>50	50

Supplementary References

1. Zhao Y *et al.* 2019 The dynamics of deformed wing virus concentration and host defensive gene expression after Varroa mite parasitism in honey bees, *Apis mellifera*. *Insects* **10**. (doi:10.3390/insects10010016)

2. Simone M, Evans JD, Spivak M. 2009 Resin collection and social immunity in honey bees. *Evolution* **63**, 3016–3022. (doi:https://doi.org/10.1111/j.1558-5646.2009.00772.x)

3. Evans JD. 2006 Beepath: An ordered quantitative-PCR array for exploring honey bee immunity and disease. *J Invertebr Pathol* **93**, 135–139. (doi:10.1016/j.jip.2006.04.004)

4. Wang Y, Qian P-Y. 2009 Conservative fragments in bacterial 16S rRNA genes and primer design for 16S ribosomal DNA amplicons in metagenomic studies. *PLoS One* **4**, e7401. (doi:10.1371/journal.pone.0007401)
